# Association of fronto-temporal function with cognitive ability in schizophrenia

**DOI:** 10.1038/srep42858

**Published:** 2017-02-16

**Authors:** Shenghong Pu, Kazuyuki Nakagome, Masashi Itakura, Masaaki Iwata, Izumi Nagata, Koichi Kaneko

**Affiliations:** 1 Division of Neuropsychiatry, Department of Brain and Neuroscience, Tottori University Faculty of Medicine: 36-1 Nishi-cho, Yonago, Tottori 683-8504, Japan; 2 National Center of Neurology and Psychiatry: 4-1-1 Ogawa-Higashi, Kodaira, Tokyo 187-8551 Japan

## Abstract

Deficits in neuropsychological performance are common in schizophrenia, but their relationship with the fronto-temporal functional abnormalities associated with this condition remains unclear. We explored the relationship between neuropsychological performance as measured using the Brief Assessment of Cognition in Schizophrenia (BACS) and the Social Cognition Screening Questionnaire theory of mind (ToM) subscale and fronto-temporal function in 23 patients with schizophrenia and 23 age- and gender-matched healthy controls (HCs), using 52-channel near-infrared spectroscopy (NIRS). Regional hemodynamic changes were significantly smaller in the schizophrenia group than in the HCs group in the ventro-lateral prefrontal cortex and the anterior part of the temporal cortex (VLPFC/aTC) and dorso-lateral prefrontal cortex and frontopolar cortex (DLPFC/FPC) regions. To dissect the effect of variance in BACS cognitive domains from the relationship between ToM function and fronto-temporal function, we performed additional partial correlation analyses between ToM and NIRS data, using BACS composite score as a control variable. The correlation between ToM and NIRS data remained significant only in the DLPFC/FPC region. This finding is important to models of recovery, as it suggests that intervention programs focusing on enhancing fronto-temporal function may have a greater impact on social and occupational outcomes than traditional rehabilitation programs focusing on neuropsychological performance.

Cognitive impairments are recognized as core functional deficits of schizophrenia and are a major factor in the unsuccessful re-entry of patients with schizophrenia into the community after treatment^1^,^2^. Schizophrenia is associated with deficits in multiple cognitive domains, including the neurocognitive domains (selective and sustained attention, working memory, episodic memory, processing speed, executive function) and the social cognitive domains^2^. Because of the lack of effective treatments for these deficits, schizophrenia often continues to exact a devastating toll, with 80% of patients remaining unemployed and less than 30% living independently^3^.

Neurocognitive and functional disabilities are credible treatment targets^4^. The association between improved neurocognitive skills and improved functional outcomes has led to the implementation of neurocognitive remediation programs aimed at improving an individual’s cognitive skills, and thereby, functional recovery and real world community outcomes^5^. However, the current evidence for the impact of such programs on real-world improvements in functional status is equivocal^6^.

Schizophrenia involves dysfunction of the prefrontal cortex (PFC) via several mechanisms, such as perfusion defects, defective metabolism, and flawed synaptic pruning. The PFC is responsible for higher cognitive function (such as planning, regulation, and execution of goal-directed activities) and motivation^7^,^8^,^9^. In humans, functional magnetic resonance imaging (fMRI)^10^,^11^,^12^,^13^ and near-infrared spectroscopy (NIRS) imaging^14^ has shown that incentivized conditions also amplify PFC activity associated with working memory. Thus, the functional significance of the PFC may lie in the integration of motivational and cognitive operations for goal-directed behaviour.

In recent years, social cognitive ability has garnered attention in schizophrenia research because of its relationship to functioning^15^ and its potential as a proximal target for treatment aimed at improving social functioning^16^. In examining the overlap of neurocognitive and social cognitive impairment, the majority of participants in a sample of 119 psychiatrically stable outpatients with schizophrenia (68%) were impaired in both these domains^17^.

Existing research indicates that: 1) both neurocognitive and social cognitive abilities are impaired in schizophrenia and predict the level of functioning^18^,^19^; 2) social cognitive impairments appear to be present in schizophrenia across different phases of the illness^20^,^21^; and 3) neurocognitive and social cognitive domains correlate in the medium-range^22^,^23^. Results of a recent meta-analysis indicate that social cognitive and neurocognitive domains generally correlate in the 0.22 to 0.33 range^24^. Penn and colleagues^25^ suggested that neurocognitive ability may represent a “necessary but not sufficient” prerequisite for social cognitive ability (p. 115). Having found that good social cognitive ability is extremely rare in the presence of poor neurocognitive ability, and that the reverse is far more common, we may speculate that neurocognitive ability form a “necessary” basis of social cognitive ability^17^.

Although neurocognitive function has been closely associated with social functioning, a recent view has proposed that multiple mediators, including social cognitive ability, beliefs, and motivation, exist between the 2 components^26^. When these mediators are included in the path model, the relationship between neurocognitive function and social functioning is no longer straightforward^27^,^28^,^29^. In fact, working memory-related PFC activity may represent one of these mediators. Therefore, elucidating the relationship between working memory-related PFC neural activity and cognitive ability could help to clarify the mechanism(s) of schizophrenia-related cognitive dysfunction. However, while remediating or compensating for neurocognitive dysfunction is an important consideration in the refinement or design of future interventions targeting the social cognitive ability, ameliorating neurocognitive deficits in and of itself may not be sufficient to improve social cognitive performance. Thus, continued efforts in designing and evaluating social cognitive interventions are required, as are further analyses of non-cognitive variables that may be related to social cognitive impairments in schizophrenia^17^.

Our goals in the present study were to test the following hypotheses. (1) Relative to healthy subjects, patients with schizophrenia have detectable abnormalities in working memory-related PFC neural activity, as measured using multi-channel NIRS imaging. (2) This neural activity is related to cognitive ability, in particular ToM (see our previous study^30^), and working memory or verbal memory, and verbal fluency cognitive domains, as the improvement in these subcomponents in BACS was associated with the increased hemodynamic response caused by cognitive remediation therapy in our previous study^31^. In the present study, we analysed regional activation associated with cognitive tasks in 3 frontal brain regions of interest (ROIs) defined by Takizawa *et al*.^32^ (see Fig. 1). Findings supporting these hypotheses would suggest that working memory-related PFC neural activity may be a useful biomarker for therapeutic development.

## Results

The participants’ demographic data are shown in Table 1.

### Task performance

The response sensitivity A′ and reaction time (RT) scores in the 2-back task during NIRS measurement were significantly worse in the schizophrenia group than in healthy controls (HCs) (Table 1).

### Cognitive activation

The overall mean oxy-Hb changes waveforms for the 52 channels and the 3 ROIs in each group are shown in Fig. 2. Patients with schizophrenia exhibited significantly smaller integral values of oxy-Hb changes than Hcs (region 1: p = 0.048; left region 2: p = 0.001; right region 2: p = 0.000). The between-group differences for integral values of oxy-Hb changes remained significant after correcting for performance levels in 3 ROIs (region 1: p = 0.036; left region 2: p = 0.008; right region 2: p = 0.005) as assessed by ANCOVA using RT and sensitivity A′ as covariates of the integral values of oxy-Hb changes.

### Correlation Analyses

In patients with schizophrenia, bivariate Pearson correlations (r) between cognitive function and integral values of oxy-Hb changes are presented in Table 2. Significant correlations of both cognitive function dimensions were found with the change in integral values in the left and right region 2. Only the ToM scores (r = 0.463; p < 0.05) correlated significantly with integral value changes in region 1, while the correlation with the BACS composite scores (r = 0.179; p > 0.05) did not reach significance (Table 2).

Although neurocognitive and social cognitive abilities segregate as independent dimensions in factor analytical studies, they co-occur in patients with schizophrenia^17^. In the current study, the correlation was r = 0.635 (p = 0.001). We applied partial correlations to independently assess the dimensions relation with the integral value of oxy-Hb changes in the region 1 and region 2 (see Table 2 coefficients in brackets). None of the integral value oxy-Hb change indices were related to the BACS composite scores if the variation in ToM level was held constant. However, when the BACS composite scores were held constant, ToM scores significantly correlated with the integral value of oxy-Hb changes specifically in region 1, which was significantly different from that between the BACS composite scores and oxy-Hb changes in region 1 (z = 2.11, p < 0.05).

## Discussion

In this study, we used multi-channel NIRS to identify a relationship between working memory-related hemodynamic responses in fronto-temporal regions and neuropsychological performance as measured using the BACS and the ToM in patients with schizophrenia. At the level of working memory-related hemodynamic responses, we found that activity in the bilateral VLPFC/aTC (left and right region 2 in the present study) were significantly associated with both these cognitive function dimensions, while only the ToM scores (r = 0.463; p < 0.05) were significantly correlated with the DLPFC/FPC (region 1 the present study). When we controlled for variation in the other dimension, working memory-related hemodynamic responses specifically in region 1 showed significant correlation with ToM scores which was significantly different from that between the BACS composite scores and oxy-Hb changes. None of the partial correlations between the BACS scores and working memory-related hemodynamic responses in fronto-temporal regions (region 1, left and right region 2) reached statistical significance.

Recent studies have assessed the neurocognitive domains together with the social cognitive domains in clinical rating scales, reporting mixed results regarding their link to brain structure and function^30^,^31^,^33^,^34^,^35^. Critically, none of these studies investigated the relationship of these 2 cognitive dimensions with brain structure and function independently. When we considered this in the present study, we found a dominant role for ToM in activating the working memory-related hemodynamic responses in the DLPFC/FPC regions.

The DLPFC maintains goal-relevant information during working memory^36^,^37^,^38^, updates this information as goals dynamically change during task-switching^12^,^39^,^40^,^41^, and arbitrates between conflicting goals during decision making^41^,^42^,^43^,^44^. Evidence has suggested that the DLPFC is involved in processing cognitive ToM (e.g., the false-belief task)^45^,^46^. A very recent report has shown that ToM performance was significantly lower in patients with DLPFC damage than in their control group counterparts^47^. Thus, it seems reasonable to consider that DLPFC functioning is relevant to ToM performance, above and beyond its relevance to traditional working memory^30^.

The FPC region plays a key role in coordinating and integrating information from other PFC regions^48^,^49^. Recently, Bludau *et al*.^50^ suggested that the lateral FPC region, which we investigated in the current study, is responsible for abstract cognitive behaviour, including organized conduct, action planning, and multi-goal management. Furthermore, lesion studies have highlighted the key role of prefrontal and frontal brain areas in ToM abilities^51^,^52^,^53^,^54^,^55^. Together, these findings indicate that the hemodynamic response in the DLPFC/FPC regions during a working memory task may be associated with ToM function in patients with schizophrenia. The functional properties of the DLPFC/FPC are particularly important from a clinical point of view, in that they may provide hints as to how ToM function can be improved in subjects with schizophrenia.

In addition, the hemodynamic responses correlated positively with the three domains of the BACS (working memory: left region 2; motor speed: left and right region 2; verbal fluency: right region 2) in the VLPFC/aTC regions in patients with schizophrenia. Impairments in working memory and other cognitive functions are cardinal neuropsychological symptoms in schizophrenia, and the PFC is important for mediating and executing these functions^56^,^57^,^58^. Hence, dysfunctional information processing in the PFC could be, at least to some extent, accountable for WM and other cognitive deficits in schizophrenia^34^,^59^,^60^. Our results suggest that working memory-related PFC neural activity may be a useful biomarker for therapeutic development for cognitive dysfunction in schizophrenia.

Though it seems certain that the PFC contribute to multiple cognitive functions, it remains unclear how these functions should be separated and defined. Deficits in a variety of neurological and neuropsychiatric conditions are entirely explained by loss of fluid intelligence. Partialling out the general effects of fluid intelligence, we propose, may clarify the role of additional, more specific cognitive impairments in conditions such as schizophrenia^61^,^62^,^63^,^64^.

Considering that good social cognitive ability is rare in the presence of poor neurocognitive ability, and that the reverse is far more common, neurocognitive ability may be a “necessary” basis of social cognitive ability^17^. Researchers conducting interventions for social cognition should consider the nature and severity of concomitant neurocognitive deficits and design the treatments in a way that reduces cognitive load or provide them in the context of neurocognitive remediation. Differential emphasis may need to be placed on remediating specific domains of neurocognitive ability depending on the social cognitive domain targeted by an intervention^17^, which should be clarified in future studies.

Although ToM was broadly associated with working memory-related hemodynamic responses in the DLPFC/FPC regions, these preliminary results should be interpreted within the context of the study limitations. This was a cross-sectional investigation, and because ToM was not observed over time, inferences regarding the causality of the association between ToM and working memory-related hemodynamic responses in the DLPFC/FPC regions cannot be made. Second, this investigation involved a relatively small sample size (N = 23), which may have hampered our ability to detect lesser relationships between ToM and working memory-related hemodynamic responses in the fronto-temporal regions. Further studies with a larger sample size are required to verify our findings. Third, although we did not find any relationships between oxy-Hb signals and the duration of illness or dosages of medication in patients with schizophrenia, most of the patients in this study were chronically ill and were medicated. Thus, to rule out medication effects comprehensively, future studies are warranted using first-episode and/or drug-naïve patients with schizophrenia. Fourth, the sample was obtained from the outpatient population of Tottori University hospital and the number of male and female patients differed markedly, and thus may not be considered to be representative of the general population of patients with schizophrenia. Fifth, we did not administer the SCSQ in normal controls, although in a previous study, we found a reliable discriminatory effect between patients and normal controls using SCSQ total scores and ToM subscale scores^65^. It is also crucial to investigate whether the same association could be found in normal control populations to clarify whether the present finding is specific to patients with schizophrenia or is much more common. Lastly, even though NIRS is a method well-suited for obtaining physiological data about the cerebral cortex, it was not possible to determine the definite locations of cortical regions comprehensively. Although the ToM subscale score in the SCSQ was assumed to rely more on the cognitive processing of ToM, it was considered inappropriate for disentangling the locations between the DLPFC/FPC regions. Therefore, different PFC areas might have partly contributed to the observed association. In future studies, it would be appropriate to add other ToM tasks that are more relevant to affective processing, such as the faux pas task, to distinguish the role of the PFC in cognitive versus affective components of ToM.

In conclusion, despite these limitations, our study indicated that impaired ToM function may be related to DLPFC/FPC function in patients with schizophrenia. Our findings extend our knowledge about potential mechanisms underlying ToM function and have implications for the treatment of these patients. In future, these findings should be verified by further studies with a larger sample size.

## Material and Methods

### Participants

The study was approved by the Ethics Committee of the Faculty of Medicine of Tottori University, Tottori, Japan, and the investigation was carried out in accordance with the latest version of the Declaration of Helsinki. All participants gave informed written consent.

The schizophrenia group consisted of 23 patients (7 male, 16 female) who had been diagnosed with schizophrenia based on the Diagnostic and Statistical Manual of Mental Disorders, fourth edition (DSM-IV), using the Mini-International Neuropsychiatric Interview (MINI)^66^. The patients were recruited from both outpatient and inpatient services at the Tottori University Hospital. The details of these patients are given in Table 1. On the day of the NIRS experiment, psychiatric symptoms were evaluated by the same psychiatrists (I.N., and K.K.) using the Positive and Negative Syndrome Scale (PANSS)^67^. Patients with comorbid neurological illness, previous traumatic brain injury with any known cognitive consequences or loss of consciousness for more than 5 min, a history of electroconvulsive therapy, or alcohol/substance abuse or addiction (except nicotine) were excluded.

Healthy individuals who were appropriately age- and gender-matched to the schizophrenia patients participated as controls in the present study. Inclusion criteria for controls were similar to those for the patient sample, although controls were additionally required to have no previous or current psychiatric illnesses. Twenty-three individuals (7 male, 16 female) meeting these criteria were selected as participants. In the present study, healthy controls participated in the NIRS measurement but not in the cognitive assessment using BACS and SCSQ.

All patients were receiving atypical antipsychotic medication. Daily doses of all antipsychotics were converted to the equivalent dose of chlorpromazine^68^. Premorbid intelligence quotient (IQ) was estimated using the Japanese version of the National Adult Reading Test^69^. All participants were right-handed according to the Edinburgh Handedness Inventory^70^ and were native Japanese speakers.

### Cognitive measures

Brief Assessment of Cognition in Schizophrenia (BACS).

We used the Japanese version of the Brief Assessment of Cognition in Schizophrenia (BACS)^71^,^72^ to assess the neurocognitive domains. The cognitive domains included in the BACS are verbal memory, working memory, motor speed, verbal fluency, attention, and executive function. The primary measure from each BACS test was standardized by creating z-scores, in which the mean score of Japanese healthy controls was set to zero and the standard deviation was set to one^73^. A composite score was calculated by averaging the z-scores of the 6 primary measures mentioned above. Typically, when BACS is used, the average value is used as a composite score. Exploratory factor analysis of BACS subtest scores indicated a single-factor solution^74^. Thus, the BACS assesses a similar unitary cognitive construct, which is well measured by the test’s standard composite score obtained by averaging the subscale scores. Table 3 presents the correlations among the BACS primary measures and composite score. The BACS composite score was significantly correlated with all primary BACS measures. The BACS thus enables an assessment of overall cognitive function as well as provides scores on individual cognitive domains^71^,^72^.

The Social Cognition Screening Questionnaire (SCSQ).

The SCSQ^65^,^75^ contains 5 subscales, i.e., verbal working memory, schematic inference, theory of mind (ToM), metacognition, and hostility bias (range: 0–10, with higher scores indicating better performance).

### NIRS methodology

A 52-channel NIRS (ETG-4000, Hitachi Medical Co., Tokyo, Japan) machine was used to measure relative changes in oxygenated hemoglobin (oxy-Hb) and deoxygenated hemoglobin (deoxy-Hb) at 2 wavelengths (695 and 830 nm) of infrared light, based on the modified Beer–Lambert law^76^. The probes of the NIRS machine were placed on the fronto-temporal regions of each participant, with the midcolumn of the probe located over Fpz and the lowest probes located along the T3−Fp1−Fpz−Fp2−T4 line, in accordance with the International 10–20 System used in electroencephalography. This probe arrangement enabled the measurement of Hb values from the surface regions of both the PFC and temporal regions. The correspondence between the NIRS channels and cortical anatomy has previously been confirmed in a multi-subject study^77^. Spatial information from each channel was estimated using functions from the Functional Brain Science Laboratory at Jichi Medical University in Japan (http://www.jichi.ac.jp/brainlab/virtual_reg.html)^78^.

The sampling frequency was 10 Hz. To examine 2-back task-related activation, data were analyzed using the “integral mode” installed on the NIRS machine, in which the pre-task baseline was calculated as the mean over the 10-s period immediately prior to the task period, and the post-task baseline was calculated as the mean over the 5-s period that followed after the 50-s post-task period. Linear fitting was applied to the data recorded between these 2 baselines. A moving-average method, using a 5-s window, was applied to remove any short-term motion artifacts. In addition, we rejected noise related to body-movement artifacts (no signal, high frequency, and low frequency) using the algorithm published by Takizawa *et al*.^32^.

### N-back task

**Figure 1:**
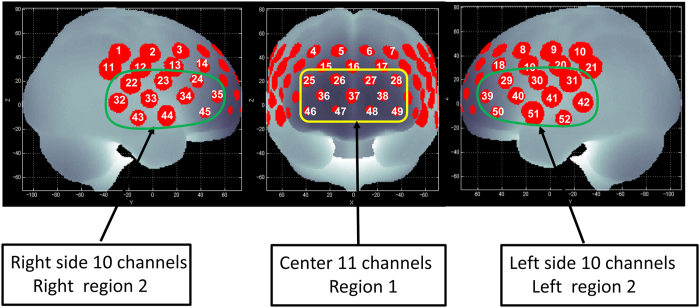
Channel positions on the brain surface. Right region 2 consists of the right 10 channels (22–24, 32–35, and 43–45) and is located approximately on the right ventro-lateral prefrontal cortex (VLPFC) and anterior part of the temporal cortex (aTC) region. Left region 2 consists of the left 10 channels (29–31, 39–42, and 50–52) and is located approximately on the left VLPFC and aTC region. Region 1 consists of the center 11 channels (25–28, 36–38, and 46–49) and is located approximately on dorso-lateral prefrontal cortex (DLPFC) and frontopolar cortex (FPC) region.

**Figure 2:**
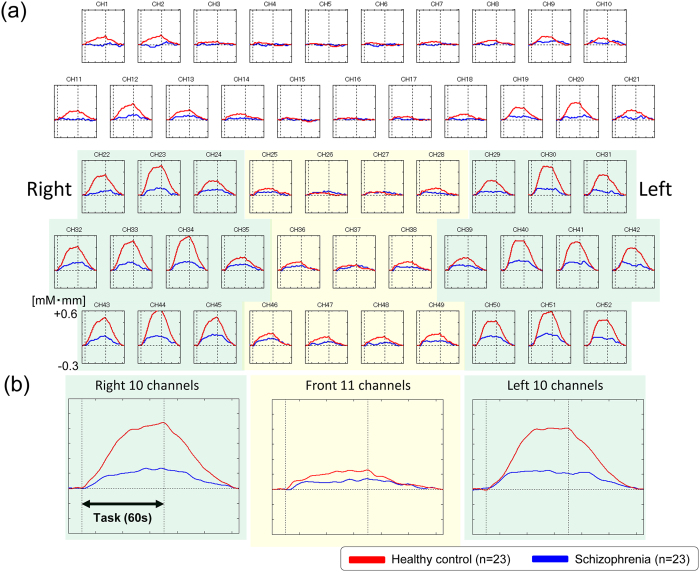
Waveforms of oxy-Hb concentration changes during the working memory task. (**a**) The upper figures show the overall average waveforms of concentrations of oxygenated hemoglobin (oxy-Hb) changes during the working memory task in patients with schizophrenia (blue) and healthy controls (red). (**b**) In the lower three figures, the right figure shows the average waveforms of oxy-Hb changes for the 10 channels in the right VLPFC/aTC region, the center figure shows that for the 11 channels in the frontal pole region, and the left figure shows that for the 10 channels in the left VLPFC/aTC region. Abbreviations: VLPFC: ventro-lateral prefrontal cortex, aTC: anterior part of temporal cortex.

We used a 2-back task with a blocked periodic baseline–activation–baseline design to activate brain regions specialized for maintenance components of verbal working memory^30^,^34^. Subjects were shown 2 contrasting conditions, which were visually presented in 60-s periods on a computer screen that was placed approximately 1 m away from the subjects’ eyes. During the baseline (B) period, subjects viewed a series of figures (0–9) presented one at a time, and were required to press a button with their right index finger whenever the figure “9” appeared (0-back). During the activation (A) period, subjects again viewed a series of figures (0–9), and were required to press a button with their right index finger if the currently presented figure was the same as that presented 2 trials previously (2-back, e.g., 5–1–5, but not 2-6-3-2, or 2-7-7). The working memory task consisted of a 60-s pre-task period (baseline [B] condition), a 60-s 2-back task period (activation [A] condition), and a 60-s post-task period (B condition). Each period was comprised of 25 stimuli (5 targets, stimulus duration: 1.8 s, stimulus onset asynchrony: 2.3 s). Behavioral performance for the 2-back task was monitored and measured in terms of RT to target figures and sensitivity A′^79^. Sensitivity A′ is an index of information processing ability using both the “hit rate (HR)” and “false alarm rate (FAR)” for calculation, as expressed in the equation below:

A′ = 0.5 + (HR − FAR) (1 + HR − FAR)/4HR (1 − FAR).

A high A′ implies high information processing ability. All subjects underwent a brief period of identical training to ensure that they understood the rules of the task prior to measurement.

### Region of interest (ROI)

Of our 52 NIRS channels, region 1 was defined as including channels 25–28, 36–38, and 46–49. The right side of region 2 was defined as channels 22–24, 32–35, and 43–45, and the left side of region 2 was defined as channels 29–31, 39–42, and 50–52^32^ (Fig. 1). The NIRS signal of region 1 consisted of the signals from channels located approximately in the dorso-lateral PFC and frontopolar cortex (DLPFC/FPC; i.e., the superior and middle frontal gyrus). Region 2 consisted of signals from channels located approximately in the ventro-lateral PFC and the anterior part of the temporal cortex (VLPFC/aTC)^32^.

### Statistical analyses

Statistical analyses were performed using SPSS Statistics 19.0 (Tokyo, Japan).

Hemodynamic responses during the working memory task in region 1 and left and right region 2 were assessed by the “integral value”^32^ of Hb changes. The integral value reflects the size of the hemodynamic responses during the 60-s 2-back task period. NIRS signals from each of the 3 representative regions (i.e., region 1, left region 2, and right region 2) were averaged separately for each type of Hb changes for each individual. We used the integral value of oxy-Hb (as opposed to deoxy-Hb) changes, as measured during the 2-back task, as an index of cortical activity, because oxy-Hb better reflects this activity and is better correlated with fMRI blood oxygenation level-dependent (BOLD) signals^80^,^81^ than deoxy-Hb.

Categorical variables were compared using the chi-square test. In all groups, the clinical variables that had a normal distribution were compared using *t*-tests, while the Mann–Whitney U-test was used for clinical variables that were not normally distributed. Integral value of oxy-Hb changes during the task period were compared between groups using *t*-tests. When there was a significant between-group difference in the performance level (RT and sensitivity A′), we performed additional analyses of co-variance (ANCOVA) using the performance level (RT and sensitivity A′) as covariates to the integral value of oxy-Hb changes. Bivariate relationships between the 2 cognitive function dimensions (BACS scores and ToM scores) and the integral value of oxy-Hb changes in the region (region 1, and left and right region 2) were assessed using Pearson’s correlation coefficients. Next, we computed partial correlations to assess the relationship between each of the 2 dimensions and the integral value of oxy-Hb changes of each ROI, independently, while controlling for the other dimension.

## Additional Information

**How to cite this article**: Pu, S. *et al*. Association of fronto-temporal function with cognitive ability in schizophrenia. *Sci. Rep.* **7**, 42858; doi: 10.1038/srep42858 (2017).

**Publisher's note:** Springer Nature remains neutral with regard to jurisdictional claims in published maps and institutional affiliations.

## Figures and Tables

**Table 1:** Demographics and clinical characteristics and cognitive test scores.

	Patients with schizophrenia n = 23 (Mean ± SD)	Healthy controls n = 23 (Mean ± SD)	Between-group comparison
Age, years	31.8 ± 9.1	31.0 ± 6.9	t (df = 44) = 0.310, *p* = 0.758
Gender, women/men	16/7	16/7	X^2^ = 0, *p* = 1.000
Edinburg handedness inventory (%)	97.4 ± 12.5	94.5 ± 9.0	t (df = 44) = 0.898, *p* = 0.374
Estimated premorbid IQ	98.5 ± 11.1	100.4 ± 8.8	t (df = 44) = −0.618, *p* = 0.540
Task performance (2-back):			
Reaction time (ms)	879.6 ± 268.1	639.7 ± 189.0	U = 116, *p* = 0.001
Sensitivity A’	0.874 ± 0.156	0.989 ± 0.022	U = 218, *p* = 0.039
Age at onset, years	21.8 ± 8.7	—	—
Duration of illness, years	10.0 ± 6.5	—	—
PANSS			
Total	63.7 ± 15.8	—	—
Positive	13.9 ± 4.1	—	—
Negative	18.1 ± 5.2	—	—
General psychopathology	31.7 ± 8.3	—	—
BACS			
Composite scores	−1.151 ± 0.917	—	—
Verbal memory	−1.321 ± 1.313	—	—
Working memory	−1.138 ± 1.086	—	—
Motor speed	−1.617 ± 1.515	—	—
Verbal fluency	−0.729 ± 0.868	—	—
Attention and speed of information processing	−1.319 ± 0.766	—	—
Executive function	−0.783 ± 1.533	—	—
SCSQ			
Verbal memory	8.0 ± 1.1	—	—
Schematic inference	7.6 ± 1.6	—	—
Theory of mind	6.4 ± 1.9	—	—
Metacognition	8.0 ± 2.2	—	—
Hostility bias	1.5 ± 1.0	—	—
Total	30.0 ± 3.2	—	—
Chlorpromazine equivalent dose, mg/day	485.3 ± 301.2	—	—

IQ, Intelligence Quotient; PANSS, Positive and Negative Symptom Scale; BACS, Brief Assessment of Cognition in Schizophrenia; SCSQ, Social Cognition Screening Questionnaire.

**Table 2:** Correlation coefficients between the cognitive ability (BACS scores and ToM scores) and the working memory-related hemodynamic responses in patients with schizophrenia.

	Region 1 (DLPFC/FPC)	Left region 2 (left VLPFC/aTC)	Right region 2 (right VLPFC/aTC)
BACS cognitive domains			
Verbal memory	0.008	0.322	0.369
Working memory	0.197	0.463^*^	0.327
Motor speed	0.083	0.480^*^	0.451^*^
Verbal fluency	0.036	0.351	0.450^*^
Attention and speed of information processing	0.154	0.332	0.388
Executive function	0.251	0.310	0.217
Composite score	0.179 (−0.169)	0.488^*^(0.227)	0.462^*^(0.229)
Social cognitive domain			
ToM	0.463^*^(0.460^*^)	0.535^**^(0.344)	0.485^*^(0.279)

Note: Values are Pearson correlations and partial correlation in brackets (each cognitive function dimension controlled for the one).

BACS, Brief Assessment of Cognition in Schizophrenia; DLPFC/FPC, dorso-lateral PFC and frontopolar cortex; VLPFC/aTC, ventro-lateral prefrontal cortex and the anterior part of the temporal cortex.

*p < 0.05.

**p < 0.01.

**Table 3:** Intercorrelations between BACS primary measures and composite score for patients with schizophrenia.

	BACS composite score
BACS	
Verbal memory	0.807^*^
Working memory	0.730^*^
Motor speed	0.823^*^
Verbal fluency	0.674^*^
Attention and speed of information processing	0.780^*^
Executive function	0.796^*^

BACS, Brief Assessment of Cognition in Schizophrenia.

*p < 0.0001.
